# Suboptimal breastfeeding practices are associated with infant illness in Vietnam

**DOI:** 10.1186/1746-4358-9-12

**Published:** 2014-08-01

**Authors:** Nemat Hajeebhoy, Phuong H Nguyen, Priya Mannava, Tuan T Nguyen, Lan Tran Mai

**Affiliations:** 1 Family Health International (FHI 360), Hanoi, Vietnam; 2 International Food Policy Research Institute (IFPRI), Hanoi, Vietnam; 3 Independent Public Health Consultant, Hanoi, Vietnam

**Keywords:** ARI, Breastfeeding, Diarrhea, Infant, Prelacteal feeding, Vietnam

## Abstract

**Background:**

Despite evidence supporting the importance of breastfeeding to child health, breastfeeding practices remain suboptimal in Vietnam. There is currently little evidence on the importance of breastfeeding in the prevention of morbidity during infancy in Vietnam. In order to provide country specific data for policy makers to support breastfeeding friendly policies and programs, analysis was undertaken on a cross-sectional dataset to investigate the association between breastfeeding practices and prevalence of diarrhea and acute respiratory infection (ARI) among infants aged 0–5 months.

**Methods:**

Data on socio-demographic characteristics, infant feeding practices and prevalence of diarrhea and ARI were obtained from 6,068 mother-child dyads in 11 provinces of Vietnam in 2011. Multivariate logistic regression was used to examine the associations between breastfeeding practices and child illnesses.

**Results:**

On average, the prevalence of diarrhea and ARI among infants 0–5 months was 5.3% and 24.5%, respectively. Though half of all infants were breastfed within one hour of birth, 73.3% were given prelacteal foods in the first three days after birth. Only 20.2% of children 0–5 months old were exclusively breastfed, while 32.4% were predominantly breastfed and 47.4% partially breastfed. After adjusting for confounders, early initiation of breastfeeding was associated with lower prevalence of diarrhea [adjusted odds ratio (AOR) = 0.74 (95% CI 0.58, 0.93)], while prelacteal feeding was associated with higher prevalence [AOR = 1.53 (95% CI 1.15, 2.03)]. Compared to infants who were exclusively breastfed, infants who were predominantly [AOR = 1.52 (95% CI 1.05, 2.21)] or partially breastfed [AOR = 1.55 (95% CI 1.07, 2.24)] were more likely to have diarrhea. Prelacteal feeding [AOR = 1.16 (95% CI 1.01, 1.33)] and partial breastfeeding [AOR relative to exclusive breastfeeding = 1.24 (95% CI 1.03, 1.48)] were associated with higher prevalence of ARI. While the protective effects of exclusive breastfeeding against diarrhea declined with child age, this effect for ARI appears to have remained constant.

**Conclusions:**

Early initiation and exclusive breastfeeding protects against diarrhea and ARI. Results confirm that interventions to improve early and exclusive breastfeeding would contribute to improving child health and nutrition in Vietnam.

## Background

The importance of breastfeeding to infant growth and development [1], as well as long-term health outcomes [2], are now well recognized. Apart from being a natural source of nourishment, human milk contains glycans and secretory immunoglobulin, which offer protection against infectious disease [3,4]. During the first six months of life, when digestive systems are not yet mature, exclusive breastfeeding (EBF) offers additional protection from illness by limiting exposure to contaminated foods and liquids. Substantial evidence shows that EBF for six months is associated with decreased morbidity and mortality from gastrointestinal infections, namely diarrhea, compared to other breastfeeding practices [5-7]. Infants who are exclusively breastfed for only three to four months are more likely than those exclusively breastfed for six months to suffer from conditions such as respiratory and urinary tract infections [8-12], otitis media [8,13,14], and necrotizing enterocolitis [8,15]. The protective effects of breastfeeding are more pronounced in settings with poor sanitation and hygiene, where contamination of foods and liquids is more likely [16-18].

Based on such evidence, the World Health Organization (WHO) currently recommends that infants be breastfed within one hour of birth, exclusively breastfed for the first six months, and then continue to be breastfed until they are two years or older along with appropriate complementary feeding initiated at the age of six months [19]. However, adoption of these recommendations remains low in developing countries where only 39% of infants are exclusively breastfed [20]. Vietnam is an example of a country where poor practice of EBF prevails [21].

Although breastfeeding is practiced by 98% of mothers in Vietnam [22], adherence to global recommendations on infant and young child feeding (IYCF) practices is poor. Evidence shows that EBF rates among infants aged less than six months are in the range of 8 to 17% [21,22], with rapid declines in rates over time [23-25]. Premature introduction of solids and semi-solids during the first three months of life has also been documented [24,26], while continued breastfeeding to 24 months of age remains low at 22% [27]. To our knowledge no studies to date, including the National Nutrition Survey and the Multiple Indicator-Cluster Survey, have explored the impact of such feeding practices on child illness in Vietnam. Country-specific data on the relationships between breastfeeding practices and infant infection are essential to advocate for policies supportive of early and EBF. The objective of this paper is to investigate the association of infant feeding practices with the prevalence of diarrhea and acute respiratory infection (ARI) among infants aged 0–5 months in Vietnam.

## Methods

### Data sources and study populations

This study used data from a baseline survey undertaken as part of an evaluation of Alive & Thrive (A&T), an initiative that aims to improve child nutrition by addressing suboptimal IYCF practices in Vietnam [28,29]. The survey was conducted in 2011 in 340 communes of 11 provinces where the A&T program is implemented. A two-stage cluster sampling technique was used to obtain a representative sample of 10,834 pairs of mothers and children under the age of 24 months. For each province, this involved: 1) selecting communes using population proportional to size and 2) selecting mother–child pairs using systematic random sampling. Given the focus on early and exclusive breastfeeding practices, analysis in this study was restricted to a sample of 6,068 pairs of mothers and infants under the age of six months.

### Ethics approval

Ethical approval for the survey was provided by the Institutional Review Board (IRB) of the Institute of Social and Medicine Studies in Vietnam (IORG number: 000663, FWA number: 00016762). Written informed consent was obtained from all mothers participating in the study.

### Data collection and variable definitions

Data for this study were drawn from a baseline household survey within the context of the larger evaluation of A&T’s interventions in Vietnam. This cross-sectional survey conducted in July and August 2011 was carried out in 11 provinces of 15 provinces where A&T operates. Data were collected by face-to-face interviews using a structured questionnaire. The outcomes of interest in this study were diarrhea and ARI, which were collected through maternal recall of symptoms in the two weeks prior to the survey. Diarrhea was defined as 3 or more loose stools in a 24-hour period [30], and ARI was defined as the presence of cough/cold with fever [31].

The main predictor was breastfeeding practices, which were classified and defined as per WHO indicators [32]: 1) early initiation of breastfeeding (defined as the proportion of children born in the last 24 months who were put to the breast within one hour of birth); 2) prelacteal feeding (defined as the proportion of children born in the last 24 months who were given any food or liquid other than breast milk during the first three days after birth); and 3) exclusive breastfeeding (defined as the proportion of infants 0–5 months of age who were fed exclusively with breast milk in the previous 24 hours - no foods, no liquids with the exception of medications such as drops, syrups). Two optional breastfeeding indicators were also considered in the analysis: 1) predominant breastfeeding (when the infant is given water, water-based drinks, fruit juice, ritual fluids, in addition to breast milk); and 2) partial breastfeeding (when the infant is given liquids and non-liquids such as milk, non-milk based products and other foods in addition to breast milk, non-breastfed infants were also included in this group).

Covariate variables were considered at the child, maternal, and household levels. At the child level, we adjusted for child age and gender. At the maternal level, we controlled for mother’s age, occupation, and highest level of education (primary, secondary, high school, and college or higher). At the household level, we adjusted for the household’s location, socioeconomic status (SES), and hygiene condition. Household location – urban or rural – was identified based on current administrative classifications in Vietnam [33]. SES index was calculated by principal components analysis [34], using several factors, such as ownership of property and land, household assets, housing conditions, and access to utilities. The SES index was then categorized into quintiles with the lowest quintile representing the ‘poorest’ in the population and the highest quintile representing the ‘richest’. For hygiene, we used two variables: type of toilet and sources of drinking water.

### Statistical analyses

Descriptive statistics were used to report the background characteristics and breastfeeding patterns of the study sample; percentage, mean and standard deviation (SD) are presented. Bivariate and multivariate logistic regressions were used to examine the associations between different breastfeeding practices and the prevalence of diarrhea and ARI. Practices for which associations were significant (p < 0.05) in bivariate analysis were included in the final model which controlled for confounding factors related to the child, mother, and household. All statistical analyses were carried out using the Stata 11.0 statistical package (Stata Inc., TX, USA) [35]. The cluster command in Stata was used to adjust for clustering effects at the commune level.

## Results

### Baseline characteristics

Table 1 outlines the baseline sociodemographic characteristics of the study sample. The mean age of children was 3.3 months (SD 1.6, range 0.03-5.98), and there were around the same number of boys (52%) and girls (48%). The mean age of mothers was 27.3 years (SD 5.5, range: 16.5 - 47.3), and nearly half of them (48.2%) achieved secondary school as the highest level of education. The majority of mother-child pairs (83.3%) resided in rural areas. Over 60% of study households had toilets with septic or semi-septic tanks, and nearly half relied on open well, rain or pool/lake water as their main source of drinking water.

**Table 1:** Sociodemographic characteristics of children and mothers surveyed (n = 6,068 mother-child pairs)

**Characteristic**	**Number (N)**	**Percentage or mean ± SD (range)**
Age of the child (months)	6068	3.3 ± 1.6 (0.03- 9.58)
Sex of the child		
Female	2910	48.0
Male	3158	52.0
Mother’s age (years)	6062	27.3 ± 5.5 (16.5- 47.3)
Mother’s occupation		
Farmer	2283	37.7
Other	3770	62.3
Highest level of mother’s education		
Primary school or less	692	11.4
Secondary school	2922	48.2
High school	1489	24.5
College or higher	964	15.9
Location of household		
Rural	5057	83.3
Urban	1011	16.7
Socio-economic status of household		
Richest	1294	21.3
Richer	1247	20.6
Average	1270	20.9
Poorer	1176	19.4
Poorest	1081	17.8
Type of toilet in household		
Septic/semi-septic tank	3894	64.2
One/two pit latrine	1249	20.6
No latrine/other latrine	922	15.2
Source of household drinking water		
Bottled water	647	10.6
Tap water	1272	21.0
Closed/pumped well water	1251	20.6
Open well water	1428	23.5
Rain, lake, pool, river water	1469	24.2

### Feeding practices

**Figure 1:**
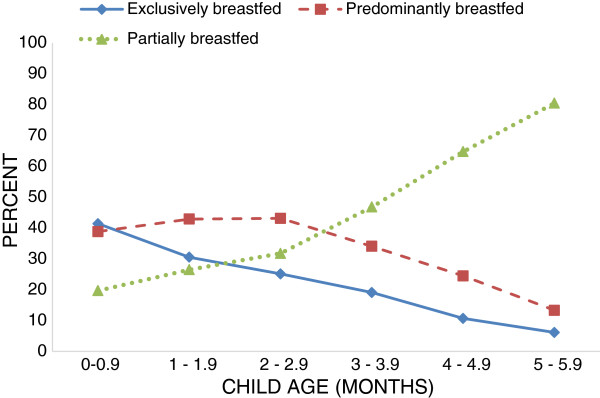
Pattern of breastfeeding practices by age (months) among infants under 6 months old (n = 6,068).

All children surveyed were ever breastfed. While half breastfed within one hour of birth, the majority of infants (73.3%) were also given prelacteal foods in the first three days after birth – namely infant formula, plain water, or honey. On average, only 20.2% of children 0–5 months old were exclusively breastfed, as opposed to 32.4% who were predominantly breastfed and 47.4% who were partially breastfed.Figure 1 provides a breakdown of breastfeeding patterns by child’s age in months. In the first month of life, 41.4% of infants were exclusively breastfed, and a similar percentage, 38.9%, predominantly breastfed. Prevalence of EBF, subsequently, declined over each consecutive month, primarily displaced by feeding of plain water, breast milk substitutes, and solid to semi-solid foods, to 6.2% by the age of 5 – 5.9 months. Around 20% of infants were introduced to solid and semi-solid foods in the first month after birth, a practice which becomes more prevalent after the age of three months: 80.5% of infants were partially breastfed at 5 months as opposed to 31.8% at 2 – 2.9 months.

### Association of feeding practices with diarrhea and ARI

**Figure 2:**
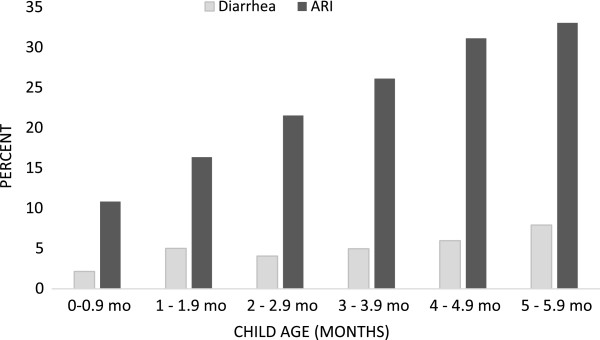
Prevalence of child illness in the previous two weeks by age (n = 6,068).

**Figure 3:**
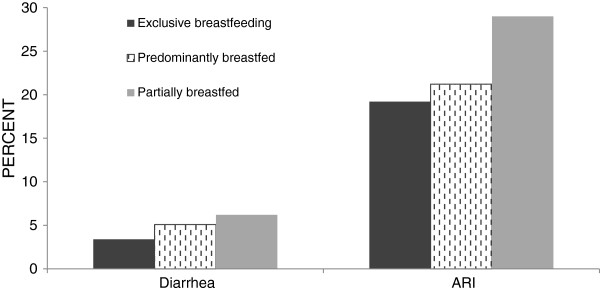
Bivariate association between breastfeeding pattern and child illness in infants under 6 months old (n = 6,068).

During the two weeks prior to the survey, 5.3% and 24.5% of all children had diarrhea and ARI respectively. The prevalence of ARI increased from 11% in the first 30 days to 33% in month five. Similarly, the prevalence of diarrhea increased from about 2% to 8% during the same period (Figure 2). Prevalence of illness varied depending on the type of breastfeeding practice (Figure 3). Table 2 presents the unadjusted and adjusted odds ratios for having diarrhea and ARI in the two previous weeks, by type of feeding practice. In bivariate analysis, early initiation and EBF were associated with lower odds of diarrhea, while the opposite was true for prelacteal feeding, predominant and partial breastfeeding. Similar results to bivariate analysis were confirmed in multivariate analysis after controlling for child, maternal, and household related factors. Adjusted odds ratios of diarrhea were 0.74 (95% CI 0.58, 0.93) for early initiation and 1.53 (95% CI 1.15, 2.03) for prelacteal feeding. Compared to exclusively breastfed infants, adjusted odds ratios of diarrhea were 1.52 (95% CI 1.05, 2.21) for predominantly and 1.55 (95% CI 1.07, 2.24) for partially breastfed children.

**Table 2:** Unadjusted and adjusted odds ratios of diarrhea and ARI in the previous two weeks by type of feeding practice, among children surveyed

	**Diarrhea**		**ARI**	
	**Unadjusted OR**	**Adjusted OR**^**1**^	**Unadjusted OR**	**Adjusted OR**^**1**^
	**(95% CI)**	**(95% CI)**	**(95% CI)**	**(95% CI)**
Early initiation of breastfeeding				
Yes	0.74*	0.74*	0.90*	0.91
	(0.59 – 0.93)	(0.58 – 0.93)	(0.80 – 1.02)	(0.80 – 1.03)
No	1.00	1.00	1.00	1.00
Prelacteal feeding				
Yes	1.48**	1.53**	1.13*	1.16*
	(1.12 – 1.95)	(1.15 – 2.03)	(0.99 – 1.30)	(1.01 – 1.33)
No	1.00	1.00	1.00	1.00
Breastfeeding status				
Exclusively breastfed	1.00	1.00	1.00	1.00
Predominantly breastfed	1.51*	1.52*	1.13	1.04
	(1.05 – 2.19)	(1.05 – 2.21)	(0.95 – 1.35)	(0.87 – 1.25)
Partially breastfed	1.86***	1.55*	1.71***	1.24*
	(1.32 – 2.62)	(1.07 – 2.24)	(1.46 – 2.02)	(1.03 – 1.48)

*p < 0.05, **p < 0.01, ***p < 0.001.

^1^Model adjusted for child (age, sex), mother (occupation, age, education), and household characteristics (location, socio-economic status, type of toilet, and source of drinking water).

**Figure 4:**
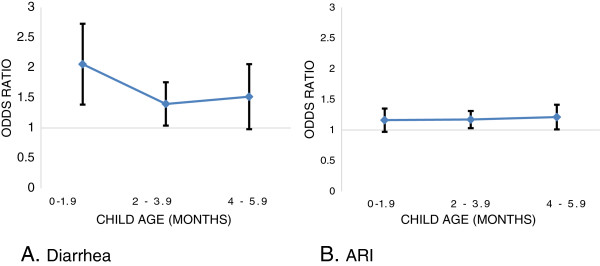
Association between the absence of exclusive breastfeeding and the diarrhea (A) and acute respiratory infection (B) by age (n = 6,068).

Breastfeeding practice was also significantly associated with ARI. Compared to exclusively breastfed infants, the odds ratio for ARI among those who were partially breastfed was 1.71 (95% CI 1.46, 2.02). This association remained significant after adjusting for confounders [AOR: 1.24 (95% CI 1.03, 1.48)]. In addition, infants with prelacteal feeding were more likely to experience ARI compared to non-prelacteal feeding counterparts (AOR 1.16, 95% CI 1.01, 1.33). While the protective effects of exclusive breastfeeding for diarrhea declined with child age, this effect for ARI appears to have remained constant (Figure 4).

## Discussion

In line with previously reported evidence, we found that breastfeeding practices in Vietnam are still sub-optimal. While all infants were ever-breastfed, a mere 20.2% were exclusively breastfed with a decreasing trend in age-specific EBF over the six-month period. This confirms that in Vietnam, the percentage of infants exclusively breastfed for the first six months continues to be among the lowest of 14 countries in the Asia Pacific region, where only two other countries report percentages below 21% [22]. We also found that only half of all infants were breastfed within the first hour, similar to the range of 35 – 49% of infants reported in a recent study on breastfeeding practices in urban and rural areas in Vietnam [25].

An overwhelming majority of infants, 73.3%, were given prelacteal foods. Liquids, solids, and semi-solids were introduced as early as immediately after birth. This is of concern as evidence shows that premature initiation of semi-solid and solid foods is associated with increased risk of nutritional imbalances and infectious disease, which in turn impact on the physical growth of the infant [24,36]. In the study on the association of complementary feeding with infant growth in Vietnam, Hop and colleagues found that infants who were partially breastfed or weaned during the first three months of life had lower weight and height gain compared to those who were exclusively breastfed [24]. Reasons cited for early introduction of complementary foods by Vietnamese mothers include concerns on the lack of sufficient breast milk production, the need to return to employment, and a lack of family support to care for the child [24,26]. This highlights the need for strengthened programs to raise awareness on early and exclusive breastfeeding, as well as for more supportive policies and workplaces for mothers.

An important finding of this study is that breastfeeding helps prevent diarrhea in infancy. Vietnam has seen rapid increases in coverage of improved drinking water and sanitation between 1990 and 2010, from 57% to 95% and 37% to 76% respectively [37]. Evidence indicates that the protective effects of breastfeeding are stronger in settings without access to hygienic water and sanitation facilities, as a result of decreased likelihood of contaminated foods and liquids [16-18]. In our study, even after controlling for several confounding factors related to the child, mother and household, the odds of diarrhea were still significantly lower among infants who were breastfed in the first hour after birth compared to those who were not. In addition, these odds were higher among infants who were predominantly or partially breastfed compared to those who were exclusively breastfed. These results suggest that the association between breastfeeding and reduced risk of diarrhea persists even in contexts with improved water and sanitation facilities.

Unlike other studies, however, we did not find that predominant breastfeeding was as protective against diarrhea as EBF – a phenomenon that has been reported in settings with hygienic water and sanitation facilities [38,39]. Rather, we found that the adjusted odds ratios for diarrhea were roughly equal for predominantly and partially breastfed infants. This suggests that during the first few months of life, providing water and water-based liquids to infants is just as detrimental as the introduction of solids and semi-solids for risk of diarrheal illness.

For ARI, we also found lower prevalence of illness among infants who were exclusively breastfed compared to those who were predominantly or partially breastfed. This difference, however, was only significant for partial breastfeeding (adjusted OR 1.24, 95% CI 1.03 – 1.48). Previous studies have documented lower prevalence of and mortality due to ARI among infants who are exclusively breastfed compared to those who are not [5,40,41]. In two Bangladeshi studies which used the same breastfeeding classifications as in this study, increased prevalence of and mortality due to ARI was also associated with partial breastfeeding only [39,42]. As was the case for diarrhea, prelacteal feeding was also associated with higher prevalence of ARI (adjusted OR 1.16, 95% CI 1.01 – 1.33), warranting the need for interventions tackling prelacteal feeding to protect infant from illness.

The stronger protective effect of optimal breastfeeding for diarrhea than for ARI may be explained by the differences in pathophysiology – gastrointestinal (GI) tract pathogens for diarrhea versus airborne pathogens for ARI. There are three pathways through which EBF can potentially protect against diarrhea: 1) limit introduction of diarrhea-causing pathogens to the GI tract; 2) immunoglobulin A in breast milk strengthens the immune system; and 3) breast milk contains glycans which function as soluble receptors that inhibit pathogens from adhering to their target receptors on the mucosal surface of the host gastrointestinal tract [3,4]. Whereas for ARI, EBF can only be protective through the boost it provides to an infant’s immune system, however, it does not reduce exposure to the types of pathogens that cause ARI.

A limitation was the study’s cross-sectional design as identification of causal relationships between breastfeeding and child illness was not possible. In addition, data on severity and duration of diarrhea and ARI were not collected. These data would have provided insights on the pathways by which protection is conferred. Use of maternal recall to measure outcomes of interest may have biased our estimates of odds ratios, but the recall period was kept to two weeks in order to limit the potential of such bias. Despite these limitations, this analysis provides important country-specific evidence for policy makers to protect, promote, and support breastfeeding. To our knowledge, this is the first analysis to examine the relationship between breastfeeding practices and child illness in Vietnam. It is based on a large sample of infants representative of several provinces in Vietnam.

In conclusion, early initiation and exclusive breastfeeding protect against diarrhea and ARI. Results confirm that interventions to increase timely and exclusive breastfeeding would contribute to improving child health and nutrition in Vietnam.

## Abbreviations

A & T: Alive & thrive; AOR: Adjusted odds ratio; ARI: Acute respiratory infection; CI: Confidence interval; EBF: Exclusive breastfeeding; IRB: Institutional review board; IYCF: Infant and young child feeding; SD: Standard deviation; SES: Socioeconomic status; WHO: World Health Organization.

## Competing interests

The authors declare that they have no competing interests.

## Authors’ contributions

NH conceived the study, participated in its design and coordination, and reviewed and revised the manuscript. PHN conceived the study, participated in its design and coordination, supervised data analysis, and drafted and revised the manuscript. PM conducted literature review and drafted the manuscript. TTN conceived the study, participated in its design and coordination and reviewed the manuscript. LMT performed the statistical analysis. All authors read and approved the final manuscript.
